# Insights Into the Role of Mitochondrial Ion Channels in Inflammatory Response

**DOI:** 10.3389/fphys.2020.00258

**Published:** 2020-04-09

**Authors:** Devasena Ponnalagu, Harpreet Singh

**Affiliations:** Department of Physiology and Cell Biology, Davis Heart and Lung Research Institute, The Ohio State University, Wexner Medical Center, Columbus, OH, United States

**Keywords:** mitochondria, inflammation, calcium, chloride intracellular channels, uncoupling proteins

## Abstract

Mitochondria are the source of many pro-inflammatory signals that cause the activation of the immune system and generate inflammatory responses. They are also potential targets of pro-inflammatory mediators, thus triggering a severe inflammatory response cycle. As mitochondria are a central hub for immune system activation, their dysfunction leads to many inflammatory disorders. Thus, strategies aiming at regulating mitochondrial dysfunction can be utilized as a therapeutic tool to cure inflammatory disorders. Two key factors that determine the structural and functional integrity of mitochondria are mitochondrial ion channels and transporters. They are not only important for maintaining the ionic homeostasis of the cell, but also play a role in regulating reactive oxygen species generation, ATP production, calcium homeostasis and apoptosis, which are common pro-inflammatory signals. The significance of the mitochondrial ion channels in inflammatory response is still not clearly understood and will need further investigation. In this article, we review the different mechanisms by which mitochondria can generate the inflammatory response as well as highlight how mitochondrial ion channels modulate these mechanisms and impact the inflammatory processes.

## Introduction

Mitochondria, largely known as the powerhouse of the cell, play a pivotal role in modulating cellular physiology (McBride et al., 2006). Mitochondria contain their own genome, including 13 polypeptide encoding genes, a small and a large rRNA gene and 22 tRNA genes (Gray et al., 2001; Andersson et al., 2003; Wallace, 2008). The mtDNA-encoded polypeptides form a core subunit of the energy generating enzyme complexes that mediate oxidative phosphorylation (OXPHOS) (Wallace, 2008). Mitochondria facilitate cellular functions such as ATP production, apoptosis (Wang and Youle, 2009), metabolism (Tzameli, 2012), calcium (Ca^2+^) homeostasis modulation (Rizzuto et al., 1992; Bernardi, 1999; Vandecasteele et al., 2001) and reactive oxygen species (ROS) generation (Murphy, 2009). As they play a central role in cellular physiology, it does not come as a surprise that mitochondrial dysfunction is observed in many pathophysiological conditions (Wallace, 2005; Nicolson, 2014). Mitochondrial dysfunction associated diseases are widespread and include neurodegenerative (Swerdlow, 2011; Karbowski and Neutzner, 2012) and cardiovascular diseases (Victor et al., 2009; Limongelli et al., 2012), diabetes and metabolic syndrome (Nicolson, 2007; Joseph et al., 2012), neurobehavioral and psychiatric diseases (Marazziti et al., 2012; Rossignol and Frye, 2012), gastrointestinal disorders (Chitkara et al., 2003), muscular atrophy (Rabinovich and Vilaro, 2010) and cancer (Wallace, 2012). Numerous studies have also shown the involvement of mitochondria in mediating the inflammatory response of immune cells (Fernandez and Perl, 2009; Vringer and Tait, 2019) and chronic infections (Gabridge, 1987; Ashida et al., 2011).

Inflammation is defined as the response generated by the immune cells upon any kind of infection or tissue injury and is a part of many physiological and pathophysiological processes (Medzhitov, 2008). A regulated inflammatory response is generally considered beneficial as it is involved in healing and the tissue repair process, as well as the prevention of further tissue or cell injury. However, this response can become detrimental if it occurs in an uncontrolled manner (Medzhitov, 2008). Several severe diseases are linked with chronic uncontrollable inflammation states (Hunter, 2012), including autoimmune disorders such as multiple sclerosis (Ghafourifar et al., 2008) and type 1 diabetes (Maiese et al., 2007). In 2005, it was demonstrated that the mitochondrial protein termed as mitochondrial anti-viral signaling (MAVS) associates with viral RNA in cases of viral infection (Seth et al., 2005). This interaction activates the nuclear factor (NF)-κB signaling pathway and eventually elicits the inflammatory response by production of type I interferons (IFN-1), such as interferon-β (Seth et al., 2005; Saitoh and Akira, 2010). In addition, it was indicated that mitochondria are a missing link in explaining the similar inflammatory responses found in two different phenomena, sepsis and systemic inflammatory response syndrome (SIRS) (Zhang et al., 2010). Zhang’s group showed that mitochondrial derived damage associated molecular patterns (DAMPs) have n-formylated peptides similar to that of bacteria and are involved in attracting neutrophils, a component of the innate immune system. These peptides were shown to activate neutrophils by binding to their surface protein – formyl peptide receptor-1 (FPR1) – to mediate the upregulation of mitogen activated protein kinase (MAPK) signaling pathways leading to chemotaxis. Thus, it was concluded that the immune response to injury/trauma mimics sepsis by mitochondrial DAMPs that activate pattern recognition receptors (PRRs) and FPR1, which are normally activated by bacterial pathogen-associated molecular patterns (PAMPs). This similarity of the inflammatory response generated by mitochondrial and bacterial peptides can be explained by the ‘endosymbiotic theory’ of mitochondrial origin (Sagan, 1967; Gray, 2017). This initial evidence indicated the potential role of mitochondria in infection by inducing an inflammatory response by immune cells.

In addition to their response to injury or infection, inflammatory processes also facilitate ‘inflammaging’ (Salminen et al., 2012), a process where accelerated aging is mediated via changes in the redox-state of the cell. The role of mitochondria in inflammaging has been extensively discussed in a recent review (Strickland et al., 2019) and hence, will not be focused on here. In this review, we will describe and discuss the various mechanisms by which mitochondria determine the generation of inflammatory responses (section “Mitochondria and Inflammatory Response”). In addition, it is well established that mitochondrial ion channels contribute to many physiological and pathophysiological processes *via* modulating the mitochondrial function (O’Rourke et al., 2005, 2007; O’Rourke, 2007; Szabo and Zoratti, 2014; Ponnalagu and Singh, 2017; Krabbendam et al., 2018; Bachmann et al., 2019). Therefore, in section “Mitochondrial Ion Channels and Inflammatory Response,” we discuss some of the mitochondrial ion channels and their significance in inflammatory responses.

## Mitochondria and Inflammatory Response

Over the years, mitochondria have emerged as playing a vital role in evoking immune responses. Mitochondrial metabolic pathways, antioxidant systems, Ca^2+^ homeostasis, mitochondrial DNA and ROS are key determinants of immune response (Angajala et al., 2018). Therefore, this section discussion is primarily focused on how mitochondrial dysfunction both at the genomic and functional level impact an inflammatory response.

### Mitochondrial Ca^2+^ and Inflammatory Response

**FIGURE 1:**
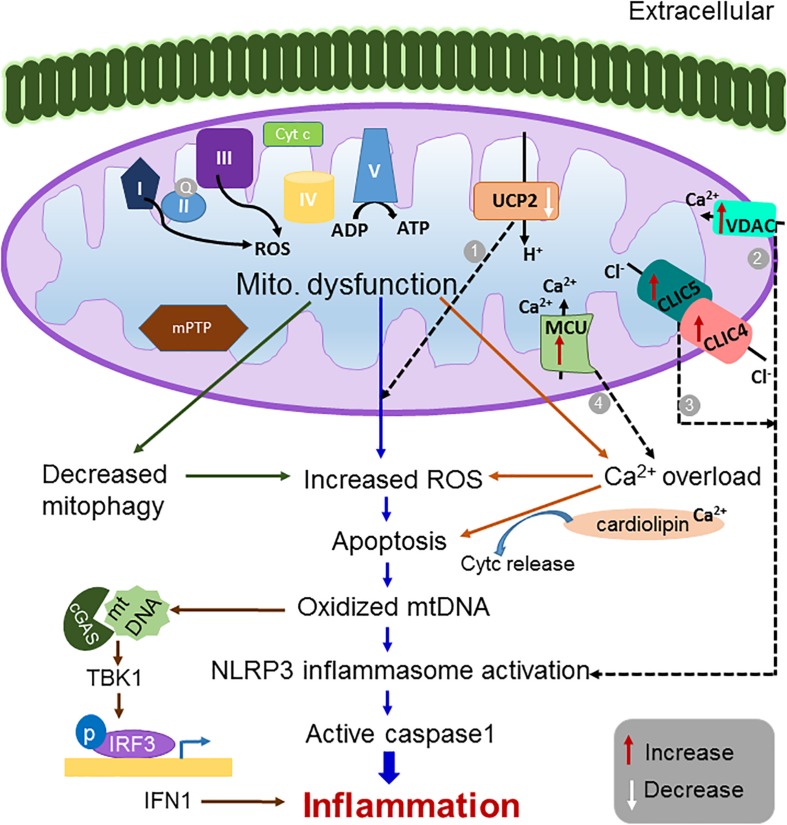
Schematic representation of the mitochondrial dysfunction leading to inflammation. Defects in mitochondrial function such as increased ROS generation, increased calcium overload, increased apoptosis and decreased mitophagy is known to activate inflammatory pathways and to manifest in several inflammatory disorders (Lopez-Armada et al., 2013). Modulation of mitochondrial ion channel expression including (1) a decrease in UCP2 (Arsenijevic et al., 2000; Vogler et al., 2006; Emre et al., 2007a; Basu Ball et al., 2011) (2) an increase in VDAC2 (Zhou et al., 2011), (3) an increase in expression of CLICs (Domingo-Fernandez et al., 2017; Tang et al., 2017), and (4) an increase in expression of MCU (Rimessi et al., 2015; Antony et al., 2016; Cheng et al., 2016) have been reported to induce excessive ROS production, NLRP3 inflammasome activation and calcium overload, respectively, thereby triggering pro-inflammatory signals. Thus, targeting these channels could be a potential therapeutic strategy for treating inflammatory disorders.

Mitochondria are known to be involved in Ca^2+^ handling (Patron et al., 2013). They are in close proximity to the endoplasmic reticulum (ER) and plasma membrane, which probably makes them respond to changes in cytosolic Ca^2+^ (Franzini-Armstrong, 2007). It is established that increased mitochondrial Ca^2+^ is a primary modulator for the production of cardiomyocyte tumor necrosis factor (TNF)-α, interleukin (IL)-1β and IL-6, leading to cardiac inflammation or dysfunction upon injury or infection (Maass et al., 2005). Interestingly, it was demonstrated that the mitochondrial Ca^2+^ exchange inhibitor, ruthenium red, decreased ROS levels, leading to reductions in pro-inflammatory mediators (Maass et al., 2005; Lopez-Armada et al., 2013). Thus, suggesting that ROS modulation could be one of the mechanisms by which mitochondrial Ca^2+^ can influence inflammatory pathways. Mitochondrial Ca^2+^ accumulation as a result of cytosolic Ca^2+^ changes is known to elevate ROS generation *via* multiple mechanisms. These include (1) activation of the tricarboxylic acid cycle (TCA), which is a driving force for the electron transport chain (ETC); and (2) stimulation of nitric oxide (NO) synthase that increases NO levels (Lopez-Jaramillo et al., 1990; Clementi et al., 1999). It was demonstrated that both exogenous addition (Brown and Cooper, 1994; Cleeter et al., 1994) and an increase in the endogenous levels of NO can bind and inhibit the ETC complexs I-IV thereby leading to increased ROS production (Clementi et al., 1999). Increased mitochondrial Ca^2+^ can bind to the oxidized state of cardiolipin (Grijalba et al., 1999; Brookes et al., 2004) and trigger the release of intermembrane space proteins, notably cytochrome c, into cytosol which then activates caspase proteases to trigger apoptosis (Wang and Youle, 2009). This results in opening of the mitochondrial permeability transition pore (mPTP), causing a loss of mitochondrial membrane potential (Ψ_*mito*_) and triggering cell death (Bernardi, 2013; Bonora et al., 2013; Giorgio et al., 2013; Bernardi and Di Lisa, 2015). Furthermore, cell death due to increased Ca^2+^ in mitochondria can also serve as a mechanism for triggering inflammatory responses independent of ROS (Figure 1).

### Mitochondria and Inflammasome Complex

Mitochondria can regulate the formation of inflammasomes (Nakahira et al., 2011; Tschopp, 2011; Zhou et al., 2011; Lopez-Armada et al., 2013). An inflammasome is a multi-protein complex which upon activation results in the stimulation of caspase-1 that in turn upregulates several inflammatory cytokines, including IL-1β and IL-18, leading to the stimulation of an inflammatory response (Strowig et al., 2012). Mitochondrial DAMPs are known to activate cytoplasmic nucleotide-binding oligomerization domain (NOD)-like receptors (NLRs). One of the well-studied NLRs showing a high association with inflammatory diseases is NLRP3. It is one of the 22 human NLR family members. Once activated, NLRP3 causes the oligomerization and recruitment of the apoptosis-associated spec-like protein containing a caspase recruitment domain (ASC) and pro-caspase 1, forming a multiprotein NLRP3 inflammasome complex (Tschopp, 2011; Lopez-Armada et al., 2013). This association is considered vital for the activation of pro-caspase 1 and downstream inflammatory events (Martinon et al., 2002). Mitochondrial ROS are capable of NLRP3 inflammasome activation (Cruz et al., 2007; Bulua et al., 2011; Zhou et al., 2011) through the inhibition of complex I or III of mitochondrial respiratory chain results in ROS generation which subsequently activates the inflammasome complex (Figure 1). Furthermore, inhibition of mitophagy/autophagy leads to spontaneous inflammasome activation due to the presence of damaged mitochondria (Zhou et al., 2011). In agreement with this, macrophages from mice lacking autophagosomal component LC3B and beclin-1 release more IL-1β and IL-18 in response to lipopolysaccharides (LPS) and ATP (Nakahira et al., 2011). Mitochondrial morphology in these knock out macrophages was altered, for example, mitochondria were swollen and produced a greater amount of ROS (Nakahira et al., 2011). An inability to remove these damaged mitochondria causes the persistence of the inflammatory states. The excessive ROS produced when not effectively resolved by the scavenging mechanism becomes a cause of uncontrolled inflammatory response in certain diseased conditions (Lopez-Armada et al., 2013). Therefore, targeting mitochondrial ROS and the NLRP3 inflammasome complex has high potential as a therapeutic agent in many types of inflammatory disease. Recently, it was demonstrated that inhibiting the enzyme choline kinase (ChoK), which is required for phosphatidylcholine synthesis, inhibits NLRP3 inflammasome activation *via* enhancing the mitophagy of damaged mitochondria (Sanchez-Lopez et al., 2019). Choline was targeted as its uptake was high within inflammatory sites, and reduction of choline uptake altered the mitochondrial lipid profile, decreased ATP synthesis and activated AMP-activated protein kinase (AMPK) (Sanchez-Lopez et al., 2019). Activation of AMPK recruits dynamin related peptide (DRP1) to mitochondria, triggering mitophagy (Sanchez-Lopez et al., 2019). Interestingly, ChoK inhibitor treatment reversed Muckle-Well syndrome, which is caused by mutation in NLRP3 genes (Sanchez-Lopez et al., 2019). As opposed to other existing drugs that can block only IL-1β, ChoK inhibitors are efficient molecular targets for inflammatory diseases as it can inhibit both IL-1β and IL-18, thus inactivating NLRP3 inflammasome (Sanchez-Lopez et al., 2019). This study further suggests that removal of damaged mitochondria could be successfully used as a therapeutic strategy to rescue uncontrollable inflammatory disease states.

### Mitochondrial DNA Mediated Inflammatory Response

The role of damaged mitochondrial DNA (mtDNA) in the inflammatory response received attention when an increased concentration of mtDNA was detected in the synovial fluid of rheumatoid arthritis (RA) patients (Collins et al., 2004). It was further shown that purified human and murine mtDNA was able to induce an inflammatory response mediated by monocytes/macrophages, but not by T or B-cells. Interestingly, neither human nor murine nuclear DNA triggered such an inflammatory response. The mechanism of inflammasome activation was attributed to unmethylated CpG motifs and oxidative damage adducts observed in mtDNA (Collins et al., 2004). In non-immune cells such as mouse primary astrocytes, it was reported that upon transfection, oxidant-initiated degraded mitochondrial polypeptides (DeMPs) induced the release of pro-inflammatory cytokines IL-6, monocyte chemotactic protein-1 (MCP-1), and TNF-α (Mathew et al., 2012). An increased expression of proinflammatory IL-1β was observed implicating the role of DeMPs in inflammasome activation. This study indicated degraded mtDNA was a new subtype of mitochondrial DAMPs possibly involved in neurodegeneration (Mathew et al., 2012) *via* activating an inflammatory response. Although several studies suggested that damaged mtDNA was necessary for inflammasome activation, the mechanism of how damaged mitochondria mediated these pro-inflammatory effects was not clear. A recent study shed light on this and showed that the binding of newly synthesized mtDNA to NLRP3 inflammasome is necessary for its activation (Zhong et al., 2018). To elucidate what triggers new mtDNA synthesis, authors found that upon binding to its target receptor TLR4, NLRP3 activators such as LPS initiate the expression of enzyme cytidine monophosphate kinase 2 (CMPK2), a rate-limiting enzyme that supplies dNTPs for mtDNA synthesis. The synthesis of mtDNA after the engagement of TLRs was concluded to be a pre-requisite for initiating NLRP3 signaling, as the macrophage lacking CMPK2 did not exhibit any inflammasome activation (Zhong et al., 2018). In addition, it was demonstrated that the new mtDNA fragments are oxidized by ROS. Contradictory to earlier reports, this study shows that even oxidized nuclear DNA exhibited these phenomena (Zhong et al., 2018), suggesting that “oxidized” DNA might be the key signal for this effect. Although this study provided a new concept of how mtDNA affects the inflammasome activation, there are still some unanswered questions, such as: (1) are only the newly synthesized mtDNA prone to oxidation by ROS? and (2) what triggers the ROS production that oxidizes the newly synthesized mtDNA? (Murphy, 2018). Nevertheless, these studies provide fascinating evidence to enhance our understanding of how mitochondria mediate the inflammatory signaling pathway and opens up a plethora of molecular targets for therapy.

mtDNA can also induce inflammatory effects via the NF-κB dependent pathway (Vringer and Tait, 2019). Some of these effects include mitochondrial DNA-dependent activation of cyclic GMP-AMP synthase (cGAS)-stimulator of interferon genes (STING), leading to an IFN-1 response (White et al., 2014). mtDNA can bind to cytosolic DNA sensors (cGAS) that upon binding to DNA produces cGAMP. The cGAMP produced would eventually bind to the ER membrane adaptor STING, and would activate it by changing the conformation of STING. This causes translocation of STING to ER-Golgi sites. During this process, it recruits and activates TANK binding kinase 1 (TBK1), which further phosphorylates the transcription factor interferon regulatory factor (IRF3). Phosphorylated IRF3s translocate to the nucleus and cause the activation of an IFN-1 response that triggers both innate and adaptive immunity (Rongvaux et al., 2014; White et al., 2014). Apart from IFN-1, the nuclear translocation of NF-κB was also observed upon mitochondrial outer membrane permeabilization (MOMP) which instigates transcription of pro-inflammatory genes (Giampazolias et al., 2017).

### Mitochondrial Cell Death Signaling and Inflammatory Response

The involvement of mitochondria in the intrinsic pathway of apoptosis indicates that mitochondria are not only the site of interaction of anti- and pro- apoptotic proteins, but also the site of signals that initiate the activation of caspases (Wang and Youle, 2009). Rupturing of the mitochondrial outer membrane by the pro-apoptotic family of proteins BAX and BAK is the initial step in triggering apoptosis. Apoptotic cells along with NF-κB signaling pathways trigger signals for the activation of NLRP3 inflammasome and the subsequent production of IL-1β. Apoptotic cells cause the release of oxidized mtDNA into the cytosol, which ultimately binds to the inflammasome complex and activates it (Shimada et al., 2012; Figure. 1). In addition to this, even silencing of the anti-apoptotic protein Bcl-2 increases IL-1β secretion, whereas Bcl-2 overexpression reverses it (Shimada et al., 2012), further showing that damaged mitochondria initiates inflammation via upregulating multiple pathways.

## Mitochondrial Ion Channels and Inflammatory Response

There is extensive literature on the mitochondrial dysfunction associated with the generation of an inflammatory response and its impact on many chronic diseases (Lopez-Armada et al., 2013; Table 1). In addition, the continuous production of ATP by mitochondria requires a huge electrochemical driving force to maintain a proton gradient across the mitochondrial membrane (Mitchell, 1966; O’Rourke, 2007). In earlier days, it was postulated that the mitochondrial membrane possesses a low- permeability of ions in order to maintain the electrochemical driving force; thus, ruling out the possibility of ion channels, transporters and exchangers in mitochondrial membranes (O’Rourke, 2007). Nevertheless, over the years, there have been identification of channels and ionic conductances in mitochondria (O’Rourke, 2007; Szabo and Zoratti, 2014; Leanza et al., 2018). Some of these channels and transporters are active under physiological conditions, whereas others get activated due to pathophysiological conditions to determine the fate of the cell (O’Rourke, 2007). Additionally, they play a key role in maintaining the ionic homeostasis in response to changes in the cytosolic ionic concentration. Mitochondrial ion channels and transporters are key regulators of redox signaling and are involved in the production of ROS and reactive nitrogen species (RNS) (O’Rourke et al., 2005; O-Uchi et al., 2014). They are also known to regulate other functions of mitochondria such as apoptosis, mitophagy and ATP production. As mitochondrial ion channels modulate multiple functions of mitochondria, they may be important in inducing inflammatory responses via the different mechanisms discussed in section “Introduction.” However, there is very little understanding of how these mitochondrial ion channels modulate immune cell activation and what role they play in regulating pro-inflammatory responses. In the subsequent subsections, we will focus on the mitochondrial ion channels and their probable role in modulating pro-inflammatory responses.

**TABLE 1:** List of Inflammatory Diseases Associated With Mitochondrial Dysfunction.

Name of the disease	Mitochondrial defects	References
Osteoarthritis and rheumatoid arthritis	Compromised mitochondrial respiration complex activities, ATP synthesis, ψ_*m*_ and increased oxidative stress	Johnson et al., 2000; Maneiro et al., 2003; Davies et al., 2008; Filippin et al., 2008; Liu et al., 2010; Biniecka et al., 2011; Lopez-Armada et al., 2013
Inflammaging	Inflammasome activation, decreased mitochondrial respiration, increased glycolysis	Franceschi et al., 2018; Strickland et al., 2019
Cardiovascular disorders and heart failure	Increased oxidative stress, increased mitochondrial Ca^2+^, mtDNA mutations	Maass et al., 2005; Garg, 2011; Oka et al., 2012; Lopez-Armada et al., 2013
Neurological disorders	Oxidative stress leading to release of pro inflammatory cytokine	Whitton, 2007; von Bernhardi and Eugenin, 2012; Lopez-Armada et al., 2013
Metabolic disorders (diabetes and obesity)	Oxidative stress, inflammasome activation	Wen et al., 2011; Martins et al., 2012; Reynolds et al., 2012; Youssef-Elabd et al., 2012; Lopez-Armada et al., 2013
Sepsis	Inhibition of mitochondrial complex activities, inflammasome activation	Apostolova et al., 2011; Zang et al., 2012; Lopez-Armada et al., 2013
Cancer	Defect in mitochondrial function of tumor infiltrating T-cells Loss of PGC1α), Alteration of T-cell function and macrophages polarization due to metabolic changes in tumor microenvironment	Fischer et al., 2007; Colegio et al., 2014; Scharping et al., 2016

### Uncoupling Proteins

Uncoupling proteins (UCPs) are integral membrane proteins located at the inner mitochondrial membrane. There are five known mammalian homologs of UCP (UCP1–UCP5) (Andrews et al., 2005; Krauss et al., 2005; Ponnalagu and Singh, 2017; Ricquier, 2017). Specifically, UCP1–UCP3 exhibit high sequence homology and are considered to be anion carriers (Busiello et al., 2015; Ponnalagu and Singh, 2017). In order to maintain the electrochemical gradient for the production of ATP, oxidative phosphorylation requires coupling of the transfer of electrons through the ETC to the pumping of protons across the inner mitochondrial membrane (Andrews et al., 2005; O’Rourke, 2007; Szabo and Zoratti, 2014). However, oxidative phosphorylation is not completely coupled, which eventually leads to the progression of ETC without ATP production (Andrews et al., 2005). This results in protons returning back to the matrix, and the energy derived from oxidation of substrates is released as heat (Andrews et al., 2005). UCP1 is the main regulator of this process in brown adipose tissue (BAT) (Nicholls, 1983), as they are predominantly present in BAT. This mechanism is important in regulating non-shivering thermogenesis in newborns, hibernating animals and over fed rodents (Oelkrug et al., 2015). In addition, UCP1 expression at both the mRNA and protein levels was also detected in some cells of white adipose tissue (WAT) which further increased upon cold or beta-adrenergic receptor agonist treatment (Cousin et al., 1992; Li et al., 2005). The UCP1-expressing cells in WAT are termed as “beige” or “brite” adipocytes because of their morphological and functional similarity to brown fat (Walden et al., 2012; Wu et al., 2012; Shabalina et al., 2013). Interestingly, UCP1-independent thermogenic mechanisms were also demonstrated in beige adipocytes of *UCP1*^–/–^ mice (Ukropec et al., 2006) raising the question of whether all the beige adipocytes of abdominal white fat express UCP1. In a recent report, mitochondrial patch clamping was performed in beige fat of two distinct adipocytes – inguinal and epididymal – and their thermogenic H^+^ leak (*I*_*H*_) properties were compared (Bertholet et al., 2017). It was demonstrated that the β3-adrenergic receptor agonist induced UCP1-dependent *I*_*H*_ properties in all the inguinal beige mitochondria, but only ∼15% of newly formed epididymal beige mitochondria exhibited UCP1-dependent proton leaking (Bertholet et al., 2017). This study further confirmed the existence of UCP1-positive and UCP1-negative beige fat cells and UCP1-independent mechanism of thermogenesis (Bertholet et al., 2017). Further, the possibility of additional UCP1-independent thermogenicity mechanisms as well as the function of other UCPs like UCP2 and UCP3 in mediating thermogenesis is still an open area of research.

Anion channel activity was associated with UCPs as they were shown to be involved in mitochondrial swelling (Nicholls and Lindberg, 1973). Furthermore, this activity also showed Cl^–^ conductance in reconstituted bilayers (Jezek et al., 1990; Huang and Klingenberg, 1996). Reconstitution of UCP1 in liposomes revealed chloride channel properties using patch-clamp technology (Huang and Klingenberg, 1996). The conductance was reported to be around 75 pS in symmetrical 100 mM KCl, and the channel closed at high positive potential on the matrix side of UCP. It was also reported that the channel gating switched from slow open-closure transitions to fast flickerings at holding potentials above +60 mV. Their selectivity for different anions was found to be Cl^–^ > Br^–^ > F^–^ > SCN^–^ > I^–^ > NO_3–_ > SO_4^2–^_ > HPO_4^2^_– > gluconate (Huang and Klingenberg, 1996).

The role of the UCP2 homolog in inflammatory responses and immunity was determined in 2000 (Arsenijevic et al., 2000). The expression of UCP2 was found to be higher in the spleen and isolated macrophages. Upon infection with *Toxoplasma gondii*, *UCP2*^–/–^ mice were found to be resistant to infection in comparison to wild type mice. Parasitic cysts and inflammation sites were also significantly reduced in case of *UCP2*^–/–^ mice. Moreover, macrophages from *UCP2*^–/–^ mice generate more ROS than wild type mice upon *T. gondii* infection (Arsenijevic et al., 2000). The inhibition of UCP2 resulted in increased ROS generation in macrophages (Figure 1; Negre-Salvayre et al., 1997; Emre et al., 2007b). These results indicate the potential role of UCP2 in regulating ROS generation in macrophages and further regulation of inflammatory responses. In addition, it was observed that UCP2 knocked down macrophages show an increased expression of proinflammatory cytokines (Basu Ball et al., 2011). UCP2 knock down was also associated with the pro-inflammatory response in autoimmune encephalomyelitis, a murine model of multiple sclerosis (Vogler et al., 2006). T-cell proliferation as well as B-cell response was increased in the *UCP2*^–/–^ mice. CD4 T-cells produced higher levels of pro-inflammatory cytokines, such as TNF-α and IL-2. Similar to macrophages, UCP2-deficient CD4 and CD8 T-cells demonstrated increased ROS emission (Vogler et al., 2006). In an autoimmune disease model of streptozotocin (STZ) induced diabetes, it was observed that knock down of UCP2 aggravated the disease with increased intra-islet infiltration of macrophages (Emre et al., 2007a). In comparison to wild type macrophages, *UCP2*^–/–^ macrophages showed increased IL-1β and NO secretion leading to a futher increase in NO/ROS mediated damage of β-islet cells (Emre et al., 2007a). Overall, the inflammation was stronger in *UCP2*^–/–^ mice causing a worsened disease in the mice (Emre et al., 2007a). Interestingly, pathogens have also evolved to use this strategy to manipulate the expression of UCP2 in order to respond to a host’s immune system. It was observed that *Leishmania* infection causes upregulation of UCP2, to suppress host macrophage defense mechanisms, likely to prevent a ROS-mediated inactivation of the host defense (Basu Ball et al., 2011). Overall, these results signify the potential involvement of the mitochondrial transporter UCP2 in modulating inflammatory responses *via* regulating mitochondrial ROS in both adaptive and innate immune cells.

### Voltage Dependent Anion Channels

Voltage dependent anion channels (VDAC) was the first mitochondrial ion channel to be reconstituted and studied at a single channel level (Schein et al., 1976; Colombini and Mannella, 2012). It is a well-established outer mitochondrial anion channel protein (Schein et al., 1976; Colombini, 1979; Neumann et al., 2010; Ponnalagu and Singh, 2017). There are three isoforms of VDAC in mammals (Szabo and Zoratti, 2014; Ponnalagu and Singh, 2017; Queralt-Martin et al., 2020). They are also referred to as porins (Zalman et al., 1980). Using planar lipid bilayers, the single channel conductance of all porins including the mammalian version was determined to be 4–5 nS, in symmetrical 1 M KCl solution, except that of *Paramecium* (De Pinto et al., 1987; Szabo and Zoratti, 2014). VDAC has a weak anion selectivity but can also conduct Ca^2+^ and has a potential binding site for it (Choudhary et al., 2010). VDAC is associated with cellular apoptosis. It was observed that upon binding to the Bcl2 family of proteins BAX and BAK, the size of the VDAC pore increases, which causes the escape of cytochrome c (Shimizu et al., 2000; Banerjee and Ghosh, 2004, 2006) thereby triggering apoptosis. VDAC1 is over expressed in cancer cells and silencing of it reduces cancer progression (Shoshan-Barmatz et al., 2015). It plays a role in the progression of cancer *via* its association with hexokinase 1 (HK1) and hexokinase 2 (HK2) in aerobic glycolytic cancers (Wolf et al., 2011). One of the reasons for the diverse role of VDAC1 could be due its localization in the outer mitochondrial membrane which allows it to mediates cellular functions *via* affecting mitochondrial functions.

Voltage dependent anion channel is involved in the activation of the NLRP3 inflammasome complex (Zhou et al., 2011). It was demonstrated that amongst the inhibition of all the isoforms of mammalian VDAC, VDAC1, and VDAC2 showed a reduced activation of inflammasome complexes in the presence of activators of inflammasomes such as R837, silica, alum and nigericin (Zhou et al., 2011; Figure 1). A significant reduction of caspase 1, ROS and IL1β secretion was observed in the case of VDAC1 and VDAC2 knockdown (Zhou et al., 2011). Downregulation of VDAC3 did not show any effect in modulating NLRP3 inflammasome activity. Once activated, NLRP3 inflammasome associates with the mitochondrial associated membrane (MAM) (Zhou et al., 2011). As VDAC is shown to be essential for metabolite and Ca^2+^ exchange between the MAM and the mitochondria (Colombini, 2004) there is a high possibility that VDAC is one of the potential connecting links between the interactions of inflammasome complexes with MAM and the mitochondria.

### Chloride Intracellular Channel Proteins

Chloride intracellular ion channel proteins (CLICs) are a unique class of ion channel proteins. They exist in both soluble and integral membrane forms (Ashley, 2003; Singh, 2010; Ponnalagu and Singh, 2017; Gururaja Rao et al., 2018). Unlike other ion channel proteins, they possess a single transmembrane domain (Singh, 2010; Gururaja Rao et al., 2017, 2018; Ponnalagu and Singh, 2017). They show high structure and sequence similarity to the glutathione S transferase (GST) family of proteins (Cromer et al., 2002; Singh, 2010). CLICs are conserved across different species. There are six paralogs of CLICs reported in mammals, identified as CLIC1-CLIC6, four in *Arabidopsis thaliana* (*At*DHAR1-*At*DHAR4), three in invertebrates [one in *Drosophila Melanogaster* (*Dm*CLIC) and two in *Caenorphabditis elegans* (EXC4 and EXL1)] (Littler et al., 2005, 2008, 2010; Singh, 2010; Gururaja Rao et al., 2017, 2018; Ponnalagu and Singh, 2017). Recently, a homolog of CLIC, stringent starvation protein A (SspA), was identified and characterized for its biophysical properties in prokaryotes (Littler et al., 2010; Gururaja Rao et al., 2017). As the name suggests, CLICs localize to various intracellular organelles such as the ER, mitochondria, nucleus and secretory vesicles (Duncan et al., 1997; Chuang et al., 1999; Edwards, 1999; Fernandez-Salas et al., 2002; Berry et al., 2003; Ulmasov et al., 2007; Littler et al., 2008, 2010; Edwards and Kahl, 2010; Singh, 2010; Valenzuela et al., 2013; Ponnalagu et al., 2016a, b; Ponnalagu and Singh, 2017; Gururaja Rao et al., 2018). Recent studies have shown the presence of CLIC1 in extracellular vesicles as well (Setti et al., 2015). Distribution of CLICs in these organelles contributes to their multifunctional role in modulating many key cellular functions. The physiological roles of CLICs and their pathophysiological effects are diverse and have been discussed in detail in recent reviews (Gururaja Rao et al., 2018, 2020).

Amongst the six mammalian paralogs, CLIC1, CLIC4, and CLIC5 are abundantly present in the heart (Ponnalagu et al., 2016a; Ponnalagu and Singh, 2017). CLIC1 is present in the cardiac ER (Ponnalagu et al., 2016b) whereas CLIC4 and CLIC5 localize to cardiac mitochondria. Apart from VDAC, CLIC4, and CLIC5 are the only other mitochondrial anion channels that have been identified at the molecular level. CLIC proteins auto-insert into membranes and can form functional redox-sensitive ion channels (Littler et al., 2004, 2010; Singh and Ashley, 2006). Even in the cardiac mitoplast, CLIC-like indanyloxyacetic acid-94 (IAA-94)-sensitive channel activity was observed (Misak et al., 2013), indicating their ability to form ion channels. CLIC proteins were first affinity-purified using its inhibitor IAA-94 (Landry et al., 1989). In addition, three sub conductance states were observed, which were attributed to the hetero-oligomerization of CLICs to allow them to function as ion channels (Tomasek et al., 2017). This further confirmed that CLICs form functional ion channels in mitochondria. CLICs are also involved in modulating mitochondrial ROS production. The absence of CLIC5 in cardiac mitochondria increased the rate of production and the total amount of mitochondrial ROS (Ponnalagu et al., 2016a). IAA-94-sensitive chloride channels are also involved in modulating the calcium retention capacity (CRC) of the mitochondria (Ponnalagu et al., 2019). In the presence of IAA-94, the CRC was significantly reduced and caused early onset of mPTP opening (Ponnalagu et al., 2019) suggesting the possible involvement of CLICs in mPTP opening. These studies indicate that CLICs are important for regulating mitochondrial functions.

Chloride intracellular ion channel proteins are also present in immune cells and play a major role in immune activation and inflammasome mediated generation of pro-inflammatory responses (Figure 1; Domingo-Fernandez et al., 2017; Tang et al., 2017). Upon phagocytosis, CLIC1 has been shown to translocate to the phagosome membrane and promote phagosome acidifications (Jiang et al., 2012). *Clic1^–/–^* macrophages showed impaired phagosome proteolytic capacity and ROS generation (Jiang et al., 2012). In LPS-stimulated macrophages, it was observed that CLIC4 is an early response gene transcribed via the NF-κB and IRF3 pathways (Ogawa et al., 2005). In support of the transcriptional upregulation (Ogawa et al., 2005) of CLIC4, another study showed that CLIC4 expression was significantly increased in the brain, heart, liver, lung, kidney and spleen upon LPS injection in mice (He et al., 2011). Increased production of TNF, IL-6, IL-12, and CCL5 was observed in the stable CLIC4 overexpressed macrophage cell upon exposure to LPS. In addition, *clic4^–/–^* mice were resistant to LPS mediated death and had a reduced level of cytokines, indicating CLIC4 is important for mediating immune activation. In a recent report, both CLIC1 and CLIC4 were shown to activate NLRP3 inflammasome upon LPS stimulation (Domingo-Fernandez et al., 2017). In the absence of CLIC1 or CLIC4 *via* siRNA mediated gene knockdown in bone marrow derived macrophages (BMDMs), it was observed that there was impaired transcription of IL-1β causing inactivation of the inflammasome complex (Domingo-Fernandez et al., 2017).

Apart from mitochondrial mediated activation of inflammasome complexes, potassium (K^+^) efflux outside the cell membrane also triggers its activation (Munoz-Planillo et al., 2013). All of the three CLICs (CLIC1, CLIC4, and CLIC5) have been demonstrated to act downstream of the K^+^ efflux-mitochondrial ROS pathway to mediate NLRP3 inflammasome activation (Tang et al., 2017). In this study, it was shown that blocking CLICs with IAA-94 inhibited the activation of the NLRP3 inflammasome complex. Further, it was demonstrated that in the presence of an NLRP3 agonist, LPS, nigericin promoted potassium efflux, followed by mitochondrial damage and increased ROS production. Also, inhibition of mitochondrial ROS did not alter the nigericin induced potassium efflux suggesting that ROS production is downstream of K^+^ efflux. Interestingly, increased IL-1β secretion was inhibited in the presence of the mitochondrial ROS scavenger MnTBAP, suggesting that K^+^ efflux induces mitochondrial damage leading to ROS production in order to promote NLRP3 inflammasome activation. Furthermore, it was shown that nigericin induced an increase in enrichment of CLIC1, CLIC4, and CLIC5 in the plasma membrane of BMDMs. This in turn was inhibited in the presence of a mitochondrial ROS scavenger (Tang et al., 2017). These results indicate that K^+^ efflux leads to mitochondrial damage resulting in increased ROS production that then mediates a possible translocation of CLICs to the plasma membrane, causing chloride efflux (Tang et al., 2017). The CLICs translocation to the plasma membrane was shown to enhance the inflammasome assembly, caspase-1 activation and IL-1β release (Tang et al., 2017).

CLICs modulate mitochondrial functions such as mitochondrial ROS generation (Ponnalagu et al., 2016a) and CRC (Ponnalagu et al., 2019) in cardiac mitochondria. Similar to cardiac mitochondria, CLICs might localize to mitochondria in macrophages. Thus, the role of mitochondrial CLICs in the regulation of inflammatory responses generated by macrophages *via* directly influencing mitochondrial function is still not clear and needs to be elucidated.

### Mitochondrial Calcium Uniporter

Association of mitochondria with Ca^2+^ was indicated in 1953 (Slater and Cleland, 1953). Later in the 1960s, it was demonstrated that rat kidney mitochondria can uptake Ca^2+^ which is dependent on the levels of ATP, Mg^2+^ and inorganic phosphate (Deluca and Engstrom, 1961; Vasington and Murphy, 1962). After 50 years, the major source of Ca^2+^ entry to mitochondria *via* a mitochondrial calcium uniporter (MCU) was identified by two independent groups (Baughman et al., 2011; De Stefani et al., 2011; Giorgi et al., 2018).

MCU is associated with many regulatory subunits including mitochondrial Ca^2+^ uptake (MICU1 and MICU2) and an essential MCU regulator (EMRE), which aids in its function of Ca^2+^ uptake (Perocchi et al., 2010; Mallilankaraman et al., 2012; Csordas et al., 2013; Plovanich et al., 2013; Sancak et al., 2013; Kamer and Mootha, 2014; Patron et al., 2014; Wang et al., 2014; Mishra et al., 2017). MICU1 is a Ca^2+^ sensing subunit which determines the activation of MCU (Csordas et al., 2013). In its absence, mitochondria become overloaded with Ca^2+^, resulting in increased ROS and apoptosis (Mallilankaraman et al., 2012). MICU1 and MICU2 work together to prevent Ca^2+^ uptake by the mitochondria when cytoplasmic Ca^2+^ is low (Kamer and Mootha, 2014). The other regulatory subunit, EMRE, bridges the interaction between MCU and MICUs, acting as a gatekeeper of the MCU by sensing Ca^2+^ in the matrix (Vais et al., 2016). In its absence, the MCU channel activity is lost. It is observed that MCU exhibits a 6–7 pS single channel activity and is sensitive to ruthenium red (Baughman et al., 2011; De Stefani et al., 2011; Chaudhuri et al., 2013). Serine 259 residue in the pore forming domain was shown to be sensitive to ruthenium red (Chaudhuri et al., 2013).

Over the years, the importance of mitochondrial Ca^2+^ has been very well recognized in metabolism, ATP production and modulating cell death pathways (Giorgi et al., 2018). Further, it is understood that mitochondrial Ca^2+^ influences inflammatory responses (summarized in section “Mitochondrial Ca^2+^ and Inflammatory Response”), suggesting the potential role of mediators of Ca^2+^ homeostasis in mitochondria such as MCU in impacting inflammatory responses. Interestingly, the importance of MCU in inflammatory responses became evident in cystic fibrosis (CF) patients. CF patients exhibit a severe lung inflammatory response as characterized by the increased expression of pro-inflammatory cytokine IL-1β (Rimessi et al., 2015). This is mainly because CF airway epithelial cells show an increased pro-inflammatory response to the pathogen *Pseudomonas aeruginosa* (Rimessi et al., 2015). It was demonstrated that MCU expression rises upon the infection, and MCU dependent increased mitochondrial Ca^2+^ uptake activates the NLRP3 complex, causing mitochondrial dysfunction such as increased ROS generation and apoptosis. Thus, showing that MCU-dependent mitochondrial Ca^2+^ loading triggers an exacerbated inflammatory response in CF patients (Rimessi et al., 2015). Similarly, liver specific conditional knock out of its regulatory subunit, MICU1, increased the pro-inflammatory responses post partial hepatectomy (Antony et al., 2016). Higher levels of serum IL-6 and tissue specific increases in TNF-α and NF-κB activity was observed in the knock out mice post hepatectomy (Antony et al., 2016) eventually leading to the inhibition of liver tissue regeneration (Antony et al., 2016). MCU was also shown to regulate the type I interferon response, upon viral infection *via* induction of several interferon stimulated genes (ISGs) (Cheng et al., 2016). It is established that viral infection always induces ER stress (Rosebeck et al., 2011; Saeed et al., 2011), which in turn plays a predominant role in determining the expression of proinflammatory cytokine (Hung et al., 2004; Hu et al., 2011). In this study, it was demonstrated that MCU: (1) interacts with the mitochondrial protein MAVS, and (2) this interaction is a pre-requisite for the activation of downstream events including phosphorylation of IRF3 and secretion of pro-inflammatory cytokine IFN-1β upon ER stress (Cheng et al., 2016). Further knock down of either MCU or MAVS did not elicit expression of IFN-1β, suggesting the significance of the interaction of MCU and MAVS in MAVS-mediated immune activation (Cheng et al., 2016). These results further suggest that similar to VDAC as described in section“Voltage dependent anion channels,” MCU *via* its role in regulating Ca^2+^ exchange can possibly act as a link between the MAM and the mitochondria upon outer mitochondrial membrane permeabilization (Missiroli et al., 2018).

## Other Channels and Transporters in Mitochondria

There are other channels, such as the calcium activated chloride channel (CLCC), volume regulated anion channel (VRAC), MITOK, small conductance calcium activated potassium (SK) channel and large conductance calcium and voltage activated potassium (BK) channels, that are either shown or predicted to be present in mitochondria (O’Rourke et al., 2005, 2007; O’Rourke, 2007; Dolga et al., 2013; Singh et al., 2013; Stowe et al., 2013; Szabo and Zoratti, 2014; Ponnalagu and Singh, 2017; Paggio et al., 2019). Nevertheless, their presence and significance in modulating the immune response *via* regulating the mitochondrial structure-function of immune cells is still not known and needs further attention.

## Concluding Remarks

Mitochondria are key organelles impacting cellular integrity by modulating several cell-death pathways. The functional role of mitochondria in generating inflammatory responses by regulating Ca^2+^ homeostasis, ROS generation, apoptosis and mitophagy/autophagy is well established (Figure 1). In addition, oxidized mtDNA is also involved in activating the inflammasome complex. There are various inflammatory disorders as listed in Table 1, which are associated with defects in mitochondrial structure-function, thus indicating the importance of mitochondria in immune cells physiology. Many of these mitochondrial functions are also modulated by mitochondrial ion channels in physiological and severe pathophysiological conditions (Figure 1). Although mitochondrial ion channels are very important in cellular processes, as well as in maintaining cellular integrity *via* modulating ionic homeostasis, limited information is available on their role in generating inflammatory responses. One of the reasons could be attributed to the lack of complete characterization and information on the molecular identity of mitochondrial ion channels (Ponnalagu and Singh, 2017). Some of the mitochondrial ion channels and transporters discussed above are well established in generating immune and inflammasome activation (Figure 1), whereas the roles of other mitochondrial ion channels in regulating inflammatory processes still needs to be deciphered. Furthermore, knowledge with respect to their ionic conductance in mitochondria upon severe inflammatory conditions is necessary to recognize their significance in maintaining ionic homeostasis. Understanding the role and mechanisms of mitochondrial ion channels in elucidating inflammatory response would further provide new targets for therapeutics, which can be utilized as a treatment for many severe inflammatory disorders.

## Author Contributions

DP and HS contributed to the writing and designing of the review article.

## Conflict of Interest

The authors declare that the research was conducted in the absence of any commercial or financial relationships that could be construed as a potential conflict of interest.

## Abbreviations

AMP: adenosine mono phosphate
AMPK: AMP-activated protein kinase
ASC: apoptosis-associated spec-like protein containing a caspase recruitment domain
ATP: adenosine tri phosphate
BAK: BCL2 homologous antagonist/killer
BAT: brown adipose tissue
BAX: BCL2 associated X
BK: large conductance calcium and voltage activated potassium
BMDMs: bone marrow derived macrophages
CF: cystic fibrosis
cGAS: cyclic GMP AMP synthase
CHOK: choline kinase
CLCC: calcium activated chloride channel
CLICs: chloride intracellular ion channel proteins
CMPK: cytidine monophosphate kinase
CRAC: calcium release activated calcium channels
CRC: calcium retention capacity
DAMPS: damage associated molecular patterns
DeMP: degraded mitochondrial polypeptides
DNA: deoxyribonucleic acid
dNTP: deoxynucleotide tri phosphate
DRP: dynamin related peptide
EMRE: essential MCU regulator
ENaC: epithelial sodium channel
ER: endoplasmic reticulum
FPR: formyl peptide receptor
GMP: guanosine mono phosphate
GST: glutathione S transferase
HK: hexokinase
IAA-94: indanyloxyacetic acid-94
IL: interleukin
INF: interferon
IRF: interferon regulatory factor
ISG: interferon stimulated genes
LPS: lipopolysaccharide
MAM: mitochondria associated membranes
MAPK: mitogen activated protein kinase
MAVs: mitochondrial anti-viral signaling
MCU: mitochondrial calcium uniporter
MICU: mitochondrial calcium uptake
MOMP: mitochondrial outer membrane permeabilization
mPTP: mitochondrial permeability transition pore
NF: nuclear factor
NKCC: sodium potassium chloride-2 co transporter
NLR: NOD like receptors
NO: nitric oxide
NOD: nucleotide-binding oligomerization
OXPHOS: oxidative phosphorylation
PAMP: pathogen-associated molecular patterns
PRR: pattern recognition receptors
RA: rheumatoid arthritis
RNA: ribonucleic acid
RNS: reactive nitrogen species
ROS: reactive oxygen species
SIRS: systemic inflammatory response syndrome
SK: small conductance calcium activated potassium
STING: stimulator of interferon genes
TBK: TANK binding kinase
TCA: tricarboxylic acid
TNF: tumor necrosis factor
UCP: uncoupling protein
VDAC: voltage dependent anion channel
VRAC: Volume regulated anion channel.
